# Stair climbing and incident atrial fibrillation: a prospective cohort study

**DOI:** 10.1265/ehpm.21-00021

**Published:** 2022-03-04

**Authors:** Ahmed Arafa, Yoshihiro Kokubo, Keiko Shimamoto, Rena Kashima, Emi Watanabe, Yukie Sakai, Jiaqi Li, Masayuki Teramoto, Haytham A. Sheerah, Kengo Kusano

**Affiliations:** 1 Department of Preventive Cardiology, National Cerebral and Cardiovascular Center, Suita, Japan; 2 Public Health, Department of Social Medicine, Graduate School of Medicine, Osaka University, Suita, Japan; 3 Department of Public Health, Faculty of Medicine, Beni-Suef University, Beni-Suef, Egypt; 4 Department of Cardiovascular Medicine, National Cerebral and Cardiovascular Center, Suita, Japan; 5 Public Health Division, Ibaraki Public Health Center, Osaka Prefectural Government, Ibaraki, Japan

**Keywords:** Stair climbing, Atrial fibrillation, Prospective studies, Japan

## Abstract

**Background:**

A protective role for physical activity against the development of atrial fibrillation (AF) has been suggested. Stair climbing is a readily available form of physical activity that many people practice. Herein, we investigated the association between stair climbing and the risk of AF in a Japanese population.

**Methods:**

In this prospective cohort study, we used data of 6,575 people registered in the Suita Study, aged 30–84 years, and had no history of AF. The frequency of stair climbing was assessed by a baseline questionnaire, while AF was diagnosed during the follow-up using a 12-lead ECG, health records, check-ups, and death certificates. We used the Cox regression to calculate the hazard ratios and 95% confidence intervals of AF incidence for climbing stairs in 20–39%, 40–59%, and ≥60% compared with <20% of the time.

**Results:**

Within 91,389 person-years of follow-up, 295 participants developed AF. The incidence of AF was distributed across the stair climbing groups <20%, 20–39%, 40–59%, and ≥60% as follows: 3.57, 3.27, 3.46, and 2.63/1,000 person-years, respectively. Stair climbing ≥60% of the time was associated with a reduced risk of AF after adjustment for age and sex 0.69 (0.49, 0.96). Further adjustment for lifestyle and medical history did not affect the results 0.69 (0.49, 0.98).

**Conclusion:**

Frequent stair climbing could protect from AF. From a preventive point of view, stair climbing could be a simple way to reduce AF risk at the population level.

**Supplementary information:**

The online version contains supplementary material available at https://doi.org/10.1265/ehpm.21-00021.

## Introduction

Atrial fibrillation (AF), the most common arrhythmia encountered in clinical practice, is a chief risk factor for cardiovascular diseases (CVDs) [1]. However, AF is not an inevitable disease as it has several modifiable risk factors such as hypertension, excessive alcohol drinking, smoking, and obesity [1, 2], and risk prevention approaches were shown to reduce its incidence [3].

On the other hand, the cardioprotective role of physical activity (PA) is well-documented [4]. Stair climbing provides a feasible opportunity for engaging in PA that demands no special training or equipment and is proven to improve cardiometabolic markers, fitness, body composition, blood pressure, and lipid profile [5, 6]. While evaluating the cumulative PA using questionnaires and wearable devices is not easy in epidemiological studies, stair climbing could be an indicator of PA engagement that straightforward questions can assess [7]. Still, the potential protective effect of stair climbing on the risk of AF has not been documented yet.

We, therefore, used data from the Suita Study, a prospective cohort study conducted in urban Japan, to investigate whether stair climbing could reduce the future risk of AF among Japanese people.

## Methods

We investigated a randomly selected sample of 8,360 people, aged 30–84 years, who were registered in the Suita Study. Our exclusion criteria included history of AF or atrial flutter (n = 42), no data on stair climbing (n = 361), or loss to follow-up (n = 1,382), leaving 6,575 participants for analysis. Participants were followed up until the date of AF diagnosis, death, or censoring.

As described elsewhere [2], AF was diagnosed using a standard 12-lead rest ECG per the Minnesota Codes (8-3-1, 8-3-2, 8-3-3, and 8-3-4) or AF medication during the follow-ups that were conducted every two years. Two trained physicians coded the ECG readings. Hospital records, check-ups, and death certificates were systematically reviewed to ascertain AF diagnosis.

A question in the Suita Study baseline questionnaire, collected between 1989 and 2005, was used to assess stair climbing “*In public and private buildings with stairs and escalators or elevators, what do you usually use to climb up to the height of the third floor or higher?*” The responses were “*I use stairs most of the time (Stairs* ≥*80%)*”, “*I use stairs more than escalators or elevators (Stairs 60–79%)*”, “*half-half (Stairs 40–59%)*”, “*I use escalators or elevators more than stairs (Stairs 20–39%)*”, “*I use escalators or elevators most of the time (Stairs <20%)*”. Because of the relatively small numbers of participants in the groups 60–79% and ≥80%, we merged them into one group ≥60%.

Age-and sex-adjusted differences in the proportions of participants by their stair climbing groups were calculated by logistic regression. The Cox proportional hazards models were used to calculate the hazard ratios (HRs) and 95% confidence intervals (CIs) of AF incidence for participants in stair climbing groups 20–39%, 40–59%, and ≥60% compared with their counterparts in the <20% group. We adjusted our results for AF risk factors as demonstrated in the Suita AF risk score [2]. Later, we stratified our results by risk factors that differed significantly (p < 0.05) across different stair climbing groups. We also conducted a sensitivity analysis by removing all subjects with follow-up periods <five years. SAS version 9.4 software (SAS Institute Inc, Cary, NC) was used for statistical analyses.

## Results

The stair climbing group <20% included more older adults, women, and obese than the other groups (p-value < 0.001) (Table 1). Within a follow-up period totaling 91,389 person-years (median 14.7 years), 295 participants developed AF. The incidence of AF distributed across the stair climbing groups <20%, 20–39%, 40–59%, and ≥60% as follows: 3.57, 3.27, 3.46, and 2.63/1,000 person-years, respectively. Stair climbing ≥60% compared with <20% of the time was associated with a lower risk of AF in the age-and sex-adjusted model 0.69 (0.49, 0.96). Further adjustment for personal, lifestyle, and health factors did not affect the association 0.69 (0.49, 0.98). Despite stair climbing in 20–39% and 40–59% of the time did not significantly reduce the risk of AF, the increasing use of stairs was associated with a concomitant reduction in AF risk (p trend = 0.042) (Table 2). The results did not change after removing subjects with follow-up periods <five years: the HRs (95% CIs) for stair climbing ≥60% compared with <20% in the three models were: 0.63 (0.44, 0.92), 0.66 (0.45, 0.96), and 0.65 (0.44, 0.95), respectively (Not shown in tables). When we stratified our results by risk factors that differed significantly across stair climbing groups in the descriptive analysis, no significant interactions were detected (p interactions >0.15) (Supplementary table 1).

**Table 1:** Age-and sex-adjusted characteristics of participants by stair climbing

**Characteristics**	**<20%**	**20–39%**	**40–59%**	**≥60%**	**P difference**
Frequency	1,586	1,596	1,773	1,620	–
Age ≥70 years, %	23.7	17.4	14.3	13.0	<0.001
Men, %	41.9	43.2	45.4	57.5	<0.001
BMI ≥25 kg/m^2^, %	23.1	20.9	19.4	17.5	<0.001
Smoking >20 cigarettes/day, %	9.8	8.5	8.2	11.0	<0.001
Alcohol intake ≥360 mg/day, %	12.0	13.1	13.8	16.9	0.130
Leisure physical activity, %	32.0	37.3	44.1	49.9	<0.001
Hypertension, %	35.4	31.6	34.1	30.0	0.067
Non-HDL-C >190 mg/dL, %	16.4	15.1	14.2	14.4	0.317
Cardiac murmur or valvular disease, %	2.8	2.6	2.2	1.9	0.207
Arrhythmia other than AF, %	2.8	2.2	2.4	2.6	0.961
History of myocardial infarction, %	0.9	0.9	0.6	0.6	0.120

**Table 2:** The association [HRs (95% CIs)] between stair climbing and atrial fibrillation risk

	**Stair climbing**				

	**<20%**	**20–39%**	**40–59%**	**≥60%**	**P trend**
Person-years	20,732	22,326	25,109	23,222	–
n	74	73	87	61	–
Incidence/1,000 person-years	3.57	3.27	3.46	2.63	–
Model I	1	0.94 (0.68, 1.31)	0.94 (0.69, 1.29)	0.69 (0.49, 0.96)	0.042
Model II	1	0.92 (0.66, 1.27)	0.95 (0.69, 1.30)	0.71 (0.50, 0.99)	0.074
Model III	1	0.92 (0.66, 1.27)	0.93 (0.68, 1.28)	0.69 (0.49, 0.98)	0.056

Model I: adjusted for age (<40, 40–49, 50–59, 60–69, ≥70 years) and sex

Model II: adjusted for Model I+ body mass index (<18.5, 18.5–24.9 or ≥25 kg/m^2^), smoking (non-current, current ≤20 cigarettes/day, or current >20 cigarettes/day), alcohol (non-current, current <360 ml/day, or current ≥360 ml/day), hypertension (yes or no), non-high-density lipoprotein-cholesterol (<130, 130–190, or >190 mg/dL), cardiac murmur or valvular disease (yes or no), arrhythmia other than AF (yes or no), and history of myocardial infarction (yes or no)

Model III: adjusted further for physical activity (yes or no)

## Discussion

This study indicated that frequent stair climbing could reduce the risk of AF in urban Japanese. Our results came in line with previous literature concluding a protective effect for PA against AF risk. A recent meta-analysis of 15 prospective studies, including 1,464,539 individuals, showed that PA engagement was associated with the decreased risk of AF 0.94 (0.90, 0.97) [8]. Another recent dose-response meta-analysis of 16 prospective studies, involving 1,449,017 individuals, showed that an increase of five metabolic equivalents of task-hour/week in PA was associated with the reduced risk of AF 0.992 (0.988, 0.996) [9].

The current study carried numerous strengths, such as investigating a randomly selected sample by sex and age category that represented urban Japanese people, using a prospective design with a long follow-up period to ensure temporality, and applying standardized approaches to ascertain AF.

However, several limitations should be addressed. Stair climbing was assessed using a simple self-administered question; therefore, recall bias was possible. Further, stair climbing was considered if the individual was climbing stairs to the third floor or higher; thus, if someone was climbing stairs to the first or second floors only, he/she might have been appointed to the <20% group, leading to a probable differential misclassification that might have underestimated the protective effect of stair climbing. Besides, floors vary in terms of the number of stairs. Future studies, therefore, should assess the average number of climbed stairs rather than floors. Also, stair climbing in the current study was evaluated during baseline only. It could be speculated that some participants might have changed their stair climbing behavior during the follow-up period. One more limitation is that refraining from climbing stairs could be a sign of an unhealthy lifestyle or preclinical conditions; factors that could be related to the increased risk of AF. It could also be the other way; for example, people who recently realized that they gained weight might have started to climb stairs as a consequence. However, both possibilities seem unlikely because adjusting for potential confounders and removing people with short follow-up periods did not materially affect the results.

Finally, to the best of our knowledge, this is the first study to investigate the impact of stair climbing on the risk of AF. We concluded that frequent stair climbing to the third floor or higher could be associated with a decreased AF risk. From a preventive point of view, it is probable to avoid AF by leading a healthy lifestyle. Promoting stair climbing is a promising PA initiative that was shown to carry population-level health impacts [10]. People, therefore, should be encouraged to climb stairs regularly.

## Abbreviations

AF: Atrial Fibrillation
CVDs: Cardiovascular Diseases
PA: Physical Activity

## Supplementary information

**Additional file 1: Supplementary table 1:** Multivariable-adjusted hazard ratios and 95% confidence intervals for the association between stair climbing and the risk of atrial fibrillation by major risk factors.

## Declarations

### Data availability

Available upon a reasonable request.
