# Supplementary material for: Stair climbing and incident atrial fibrillation: a prospective cohort study
Source: Environ Health Prev Med. 2022 Mar 4;27:10. doi: 10.1265/ehpm.21-00021 (PMC9093618; doi:10.1265/ehpm.21-00021)
Supplement: Supplementary file 1 — Additional file 1: Supplementary table 1: Multivariable-adjusted hazard ratios and 95% confidence intervals for the association between stair climbing and the risk of atrial fibrillation by major risk factors. [file ehpm-27-010-s001.docx]

**Supplementary table 1: Multivariable-adjusted hazard ratios and 95% confidence intervals for the association between stair climbing and the risk of atrial fibrillation by major risk factors**

|  | **Stair climbing** | | | | |
| --- | --- | --- | --- | --- | --- |
|  | **<20%** | **20-39%** | **40-59%** | ≥**60%** | **P interaction** |
| Age <60 years | 1 | 1.26 (0.65, 2.43) | 1.64 (0.89, 3.01) | 0.61 (0.30, 1.26) | 0.627 |
| Age ≥60 years | 1 | 0.85 (0.58, 1.24) | 0.71 (0.49, 1.04) | 0.71 (0.47, 1.05) |  |
| Men | 1 | 1.05 (0.68, 1.60) | 1.05 (0.69, 1.59) | 0.80 (0.52, 1.23) | 0.151 |
| Women | 1 | 0.83 (0.50, 1.39) | 0.82 (0.50, 1.35) | 0.50 (0.25, 0.97) |  |
| BMI <25 kg/m^2^ | 1 | 0.82 (0.56, 1.22) | 0.84 (0.57, 1.22) | 0.65 (0.43, 0.97) | 0.621 |
| BMI ≥25 kg/m^2^ | 1 | 1.25 (0.68, 2.28) | 1.28 (0.71, 2.31) | 0.89 (0.44, 1.80) |  |
| Never smokers | 1 | 0.83 (0.49, 1.41) | 1.09 (0.67, 1.76) | 0.50 (0.27, 0.93) | 0.895 |
| Ever smokers | 1 | 0.99 (0.65, 1.50) | 0.80 (0.52, 1.22) | 0.80 (0.52, 1.22) |  |
| Physically active | 1 | 1.30 (0.76, 2.23) | 1.34 (0.81, 2.21) | 0.91 (0.53, 1.57) | 0.214 |
| Physically inactive | 1 | 0.74 (0.49, 1.13) | 0.71 (0.46, 1.09) | 0.60 (0.37, 0.96) |  |
